# Solidarity and HIV Testing Willingness During the COVID-19 Epidemic: A Study Among Men Who Have Sex With Men in China

**DOI:** 10.3389/fpubh.2021.752965

**Published:** 2021-12-09

**Authors:** Hang Lyu, Yi Zhou, Wencan Dai, Shihan Zhen, Shanzi Huang, Lanlan Zhou, Liqun Huang, Weiming Tang

**Affiliations:** ^1^Zhuhai Center for Disease Control and Prevention, Zhuhai, China; ^2^China Medical University, Shenyang, China; ^3^Dermatology Hospital of Southern Medical University, Guangzhou, China; ^4^University of North Carolina Project-China, Guangzhou, China

**Keywords:** HIV testing, solidarity, COVID-19 epidemic, men who have sex with men (MSM), community connectedness

## Abstract

**Background:** Solidarity, such as community connectedness and social cohesion, may be useful in improving HIV testing uptake among men who have sex with men (MSM). This study aimed to evaluate the impact of solidarity on HIV testing before the coronavirus disease 2019 (COVID-19) and HIV testing willingness during COVID-19 among MSM in China.

**Materials and Methods:** An online survey was conducted to collect sociodemographic, sexual behavioral, and solidarity items' information from the participants. We first used factor analysis to reveal the principal component of the solidarity items and then used logistic regression to study the impact of solidarity on HIV testing, by adjusting the possible confounding factors, such as age and education.

**Results:** Social cohesion and community connectedness were revealed by the factor analysis. MSM with high community connectedness were more willing to undergo HIV testing before the epidemic adjusted by age [odds ratio (*OR*): 1.07, 95% *CI*: 1.01–1.13]. The community connectedness was also related to the willingness of HIV testing during the epidemic, with adjustments of 1.09 (95% *CI*: 1.03–1.15). People who did not test for HIV before the COVID-19 epidemic were more willing to have the HIV test during the epidemic, which was correlated with the community connectedness, and the *OR* value was 1.14 (95%: 1.03–1.25).

**Conclusion:** A high level of community connectedness helped to increase the HIV testing rate before COVID-19 and the willingness of HIV testing during the epidemic among MSM. Strategies can strengthen the role of the community in the management and service of MSM.

## Instruction

HIV remains an important public health issue among men who have sex with men (MSM) (1). In China, HIV prevalence reached 8.0% among MSM, which was 200 times higher than the general population (0.04%) (2). One of the reasons for the spread of HIV among MSM may be the low HIV testing rate (3). The limitation of current HIV testing approaches (4), the lack of MSM community engagement (5), and hesitancy to access facility-based services (6) limited Chinese MSM seeking HIV testing. To develop innovative tailored testing strategies for MSM, a good understanding of their behavior and willingness to get HIV testing is required.

Since December 2019, coronavirus disease 2019 (COVID-19) spread rapidly around the world. The epidemic outbreak was characterized by WHO on March 12, 2020, as a global pandemic (7). During the COVID-19 pandemic, the Chinese government took a series of measures to inhibit the epidemic. Due to these measures (i.e., quarantine, community isolation, and maintenance of social distance), routine HIV testing was affected and the need for testing were not imposed during the epidemic (8).

Solidarity, defined as a middle ground between collectivism and individualism (9), means that some individuals are associated in a community and this community connectedness association helps the individuals (10). Many items can define and measure the solidarity among MSM, such as community connectedness and social cohesion. Community connectedness reflects the desire of an individual to become a part of a larger community. Through community, people can establish a relationship of mutual influence, build a shared emotional connection, meet their individual needs, and be rewarded through their community affiliation (11, 12). The higher the community connectedness, the more benefits the social network brings to the people participating in the community. Social cohesion, also known as community participation (13), reflects the individual's sense of belonging and common identity through the close connection between the community and society (14). The distinction between community connectedness and social cohesion is that social cohesion more emphasizes the behavior or emotion of an individual, but community connectedness more reflects how the community affects the cognitive and affective behavior of an individual, such as communication with or trust in other people in the group, which is more difficult to operationalize.

In the previous study, MSM with high social cohesion had higher HIV testing rates in Eswatini (15). For example, a study found that community-based social support can increase the HIV testing rate among MSM (16). The mechanism for this situation is solidarity can promote HIV testing through the influence of social cohesion and community connectedness. However, whether this relationship is also exists in the Chinese context is still not clear.

In this study, we aimed to evaluate the impact of solidarity on HIV testing before the COVID-19 and the HIV testing willingness during COVID-19 among MSM in China.

## Methods

### Study Setting and Infrastructure Establishment

This online cross-sectional study was conducted among Chinese MSM from May 18 to June 2, 2020, when the COVID-19 epidemic in China was lessening. China started to issue comprehensive COVID-19 measures from January 23, 2020 and this date was used as a cut-off date of the two periods. Before January 23, namely, before the COVID-19 epidemic and after the day named during COVID-19 epidemic, banner ads to the online survey were placed in WeChat, a major social networking platform in China. If the participants completed the questionnaire and participated in a lottery draw, they would have the chance to get a T-shirt provided by a community-based organization. Potential participants clicked on the survey link and signed an electronic written consent form and accepted quantification screening. Exclusion criteria were: those who did not agree to participate in the survey, those under the age of 18, if birth sex was female, or those who had never had sex with men. The phone number and IP address were used to assure each participant could only attend the survey once. In addition, IP address was used to identify individuals residing in China. With an average HIV testing rate in the last 3 months among MSM (P) of 20%, a precision error (*d*) of 0.15 *P*, and a *CI* of 95%, the required sample size was expected to be 712. The study settings were performed as detailed in our previous study (17).

## Measures

### Sociodemographic and Sexual History

The information of MSM sociodemographic characteristics were collected: age and educational attainment (high school or below, college, and college or above). Sexual history included their sexual orientation (gay, bisexual, or unsure), number of male sex partners in the last 3 months, temporary sexual partners in the past 3 months (yes or no), and condom use frequency in the past 3 months (never, occasionally, usually, or always).

### HIV Testing Information Before and Over COVID-19 Epidemic

The information of HIV testing in MSM population was divided into two parts: before or during the COVID-19 epidemic. Before the epidemic, the history of HIV testing and HIV self-check relevance were collected. During the epidemic, the willingness of HIV testing, whether the HIV testing met the needs, and HIV self-check relevance were collected.

### Solidarity Measurements

The solidarity among MSM has been noted historically as a powerful factor in explaining and predicting MSM behavior (18). We adapted the scale from a standardized 10-item opinion solidarity scale to measure solidarity, and divided it into two core factors according to the factor analysis. The 10 items are shown in Supplementary Table 1. All the items were answered using a four-point Likert scale, four means “Strongly Agree,” three means “Agree,” two means “Disagree,” and one means “Strongly Disagree.” The higher the factor, the lower the solidarity. The scale of social cohesion was obtained by scoring: “If you need to borrow money, do you think your friends in the MSM circle will lend it to you?”, “Are you willing to discuss your private problems with friends in your MSM circle?”, “If you need a place to live, can you rely on your friends in your MSM circle to take you in?”, “Do you think your MSM circle is a harmonious circle?”, and “Can you trust most MSM you know?”. The scale of community connectedness was obtained by scoring: “Do you think you are part of the MSM group?”, “Is your attitude toward joining in the MSM community positive?”, “Are you proud of being part of the MSM group?”, “Do you think that as long as you work with your partner, the problems facing the MSM community can be solved?”, and “Do you think the problems faced by any MSM community are also problems faced by you?”.

### Statistical Analysis

An exploratory factor analysis (EFA) with orthogonal rotation (varimax) was conducted to identify the potential factors for solidarity. The variance of the factor is eigenvalue. In the initial factor solution, the first factor will account for the most variance, the second will account for the second highest amount of variance, and so on. According to the previous study, solidarity can be identified on the basis of the eigenvalue (>1.5) on scree plot (Supplementary Figure 1) (15). Percentages of variances were calculated. Factor loading of >│0.40│ was recognized as the item strongly associated with the identified factor (19).

The ordered logistic regression models were used to estimate the odds ratios (*OR*s) and 95%CI for HIV testing across the solidarity factors score before or during the COVID-19 epidemic. Model 1 was adjusted for age. Model 2 was additionally adjusted for education, number of temporary sexual partners in the past 3 months, and condom use frequency in the past 3 months. All the analyses were conducted using Stata 15.1. A *p* < 0.05 was regarded as statistically significant.

## Ethical Statement

Ethical approval was obtained from the Zhuhai Center for Diseases Control and Prevention.

## Results

This study was conducted between June 17, 2020, and November 12, 2020. Supplementary Table 2 shows the number of MSM people in the habit of receiving HIV testing before or during the COVID-19 epidemic. Overall, there were 808 people involved in the survey, and 731 people who met the eligibility criteria finished the survey. The participants were recruited from 135 cities in 30 provinces of China.

The participants are 18–62 years old, with a median age of 27 (24, 33) years old. The gender of this group was mostly male (94.25%, 689/731), unmarried (84.46%, 632/731), college graduates (44.19%, 323/731), with a personal income of 5142–8571 dollar/year (28.59%, 239/731), a company employee (white-collar) in occupation (34.47%, 252/731), and homosexual (81.67%, 597).

### HIV Testing Behaviors

As Supplementary Table 2 showed, before the COVID-19 epidemic, the percentage of people who had ever been tested for HIV was 64.8% (474/731), among whom 82.1% (389/474) used self-test kits to test HIV. During the 3 months of the COVID-19 pandemic, 58.4% (427/731) people mentioned they had a need for a HIV test, but only 64.9% (277/427) of those needs were met during the pandemic, while the majority of the needs were filled through HIV self-testing (84.1%, 233/277).

### Factors Related to Solidarity

Two factors were determined in the present study. As shown in Table 1, the social cohesion was loaded heavily on five items: borrowing money, privacy problem, staying home, harmony circle, and trusting MSM. The community connectedness factor was also loaded on five items: being part of the MSM group, positive attitude toward joining the MSM community, pride in the MSM group, solving effort, and shared problems with partners. The factor loading is shown in Table 1. The questionnaire is shown in Supplementary Table 1.

**Table 1:** Factor loadings for MSM cohesion derived from exploratory factor analysis MSM cohesion were identified at baseline.

**Variation**	**Factor 1**	**Factor 2**
	**Social cohesion**	**Community connectedness**
One_of_the_group		0.72
Positive_attitude_join		0.82
Pride_group		0.78
Solve_effort		0.74
Problem_same		0.61
Borrow_money	0.78	
Privacy_problem	0.71	
Stay_home	0.81	
Harmony_circle	0.74	
Trust_msm	0.72	

*Absolute values < 0.60 are excluded from the table for simplicity. The social cohesion (factor1) was loaded items as “If you need to borrow money, you think your friends in the MSM circle will lend it to you?”, “You are willing to discuss your private problems with friends in your MSM circle?”, “If you need a place to live, you can rely on your friends in your MSM circle to take you in?”, “Do you think your MSM circle is a harmonious circle?” and “You can trust most MSM you know?”. And the community connectedness (factor 2) was loaded heavily on “Do you think you are the one of the MSM group?”, “Your attitude toward join in the MSM community is positive?”, “You are pride of being part of the MSM group?”, “You think that as long as you work with your partner, the problems facing the MSM community can be solved?” and “Do you think the problems faced by any MSM community are also problems faced by you?”*.

### Relationship of HIV Testing and Solidarity Before COVID-19 Pandemic

Before the epidemic, community connectedness was associated with HIV testing (adjusted *OR* = 1.07, *p* < 0.05), adjusted by age only (95% *CI*: 1.01–1.13). When adjusted by age, education, number of temporary sexual partners in the past 3 months, and condom use frequency in the past 3 months, the *OR* = 1.06 (95% *CI*:1.00–1.13). But the social cohesion showed no relation to HIV testing before the COVID-19 epidemic (Table 2).

**Table 2:** The relationship between whether to be tested for HIV before the epidemic and the two common factors.

**Variation**	**OR**	**SEM**	***Z*** **-value**	***P*** **-value**	**95% CI**
**Social cohesion**					
Model 1	1.04	0.026	1.58	0.115	0.99–1.09
Model 2	1.04	0.011	1.69	0.092	0.98–1.03
**Community connectedness**					
Model 1	1.07	0.030	2.34	**0.019**	1.01–1.13
Model 2	1.06	0.031	2.11	**0.035**	1.00–1.13

*OR, Odds ratio; SEM, Standard Error of Mean; CI, confidence interval. The higher the factor, the lower solidarity. (very agree-totally disagree). Model 1 adjusted for age. Model 2 was additionally adjusted for age, education, number of temporary sexual partners in the past 3 months and condom use frequency in the past 3 months. P-value for trend was obtained by adjusting solidarity factors as continuous variables. P < 0.05 are bold*.

### Relationship of HIV Testing Willingness and Solidarity During COVID-19 Epidemic

The willingness of HIV testing during the epidemic was also correlated with community connectedness (*OR* = 1.09, 95% *CI*: *1.03–1.15*), adjusted by age only or age, education, number of temporary sexual partners in the past 3 months, and condom use frequency in the past 3 months (*OR* = 1.08, 95% *CI*: 1.02–1.14). The social cohesion showed no relation to HIV testing or HIV testing willingness (Table 3).

**Table 3:** The relationship between whether you want to be tested during the epidemic and the two common factors.

**Variation**	**OR**	**SEM**	***Z*** **-value**	***P*** **-value**	**95% CI**
**Social cohesion**					
Model 1	1.02	0.025	0.98	0.33	0.97–1.07
Model 2	1.03	0.026	1.29	0.20	0.98–1.08
**Community connectedness**					
Model 1	1.09	0.030	2.99	**0.003**	1.03–1.15
Model 2	1.08	0.031	2.81	**0.005**	1.02–1.14

*OR, Odds ratio; SEM, Standard Error of Mean; CI, confidence interval. The higher the factor, the lower solidarity. (very agree-totally disagree). Model 1 adjusted for age. Model 2 was additionally adjusted for age, education, number of temporary sexual partners in the past 3 months and condom use frequency in the past 3 months. P-value for trend was obtained by adjusting solidarity factors as continuous variables. P < 0.05 are bold*.

To know if HIV testing before the COVID-19 epidemic can influence the MSM testing needs during the epidemic, we further divided the people into two parts, according to people who had or not had a HIV test before the COVID-19 epidemic as shown in Table 4. MSM who did not test for HIV before the COVID-19 epidemic with higher community connectedness was more willing to get HIV testing during the COVID-19 epidemic (*OR* = 1.14, *p* < 0.05). But community connectedness did not influence those who had the HIV test before the pandemic. Additionally, social cohesion cannot influence the HIV testing willingness, no matter whether the MSM tested for HIV before the COVID-19 epidemic or not.

**Table 4:** The relationship between whether you want to be tested during the epidemic and the two common factors.

	**Didn't test HIV before**		**Had the HIV test before**	
	**(*****n*** **=** **257)**		**(*****n*** **=** **474)**	
	**OR (95% CI)**	***p*** **-value**	**OR (95% CI)**	***p*** **-value**
**Social cohesion**				
Model 1	1.05 (0.97–1.14)	0.22	0.99 (0.93–1.06)	0.80
Model 2	1.07 (0.98–1.17)	0.12	1.00 (0.93–1.06)	0.91
**Community connectedness**				
Model 1	1.14 (1.03–1.25)	**0.008**	1.03 (0.96–1.11)	0.37
Model 2	1.14 (1.04–1.26)	**0.007**	1.03 (0.96–1.11)	0.43

*Reference group is didn't test the HIV before the epidemic. OR, Odds ratio; CI, confidence interval. The higher the factor, the lower solidarity. (very agree-totally disagree). Model 1 adjusted for age. Model 2 was additionally adjusted for age, education, number of temporary sexual partners in the past 3 months and condom use frequency in the past 3 months. P-value for trend was obtained by adjusting solidarity factors as continuous variables. P < 0.05 are bold*.

## Discussion

Knowing the relationship between solidarity and HIV testing is essential in planning community-based HIV testing programs. Solidarity can be divided into community connectedness and social cohesion. In this study, we found that community connectedness, as well as than social cohesion, affected HIV testing significantly before the COVID-19 epidemic among MSM in China. In addition, we found that MSM who had a high degree of community connection were more willing to undergo HIV testing during the COVID-19 epidemic. MSM who did not test for HIV before the COVID-19 epidemic with higher community connectedness were more willing to get HIV testing during the COVID-19 epidemic. This study extended the existing literature by evaluating the factor analysis of community solidarity among the Chinese MSM, and assessing their association with HIV before and during the COVID-19 pandemic.

According to our data of factor analysis, solidarity in MSM can be divided into social cohesion and community connectedness. The HIV test or the willingness of HIV testing before or during the epidemic were correlated with community connectedness rather than social cohesion. In the study by Elise Grover, nine social cohesion items were used to measure present solidarity among the MSM in Eswatini (15). They take the solidarity indexes as a whole, instead of a part of solidarity, discussed the relationship between solidarity and HIV testing, and found solidarity promoted HIV testing. But other researchers think solidarity is composed of many sub-items, of which MSM community connectedness and social cohesion are the most studied (20, 21). In MSM, community connectedness means establishing a relationship of mutual influence, building a shared emotional connection, meeting their individual needs, and being rewarded through their community affiliation (22). The closer the community connectedness, the wider the spread of health promotion, such as HIV testing (20). Other than HIV testing, community connectedness can also influence the frequency of doctor visits among MSM (21).

To improve solidarity, especially community connectedness, the community-based organizations (CBO) should organize activities regularly to enhance the cohesion of members in the community (23). During the COVID-19 pandemic, online activities are worth promoting by CBO. At the same time, CBO should recruit as many members as possible to join in the community, which can be achieved by forming Wechat groups (24). In addition, the support of relevant government departments, such as the CDC, is also an important part of enhancing community connectedness, which include the support of financial, venue, and health material (25). Finally, the influence of community staff will also affect the connectedness of the community (26), so finding key opinion leaders is also important for health promotion.

We found people with higher community connectedness showed different associations to HIV testing willingness during COVID-19. For MSM who were not tested for HIV before the COVID-19 epidemic, the higher the degree of community connectedness, the more willing they were to get HIV testing during the COVID-19 epidemic. For MSM who had been tested for HIV before the COVID19 epidemic, the community connectedness did not affect the willingness to test for HIV during the epidemic. The absence of HIV testing before the epidemic can be regarded as a risk factor (27). MSM with strong community connectivity can better perceive the danger and encourage others to take the HIV test. MSMs with a high degree of community connectedness usually have more health knowledge, which can help them perceive risks (16, 28). The knowledge of HIV is possibly associated with community activity among MSM (28). The more MSM know about HIV, the more they are afraid of being infected.

Finally, there was no significant correlation between social cohesion and HIV testing willingness among the people who had HIV testing before the epidemic. According to our data, social cohesion cannot regulate either the HIV testing before the epidemic nor the willingness to test. Social cohesion, which reflects the sense of belonging and common identity of an individual through the close connection between the community and society, does less for HIV testing or HIV testing willingness. But social cohesion may influence other behaviors of MSM. In Côte d'Ivoire, researchers found that social cohesion is a determinant of prevalent HIV infection (29). Social cohesion can also influence gonorrhea and chlamydia testing among MSM (30).

There are some limitations to this study. First, we measured solidarity by self-reported data, which may cause reporting bias. More objective measurements and more robust research is necessary to evaluate the community connectedness and social cohesion of MSM individuals in the future. Second, we only focused on the willingness of HIV testing during the epidemic, but the testing result may be useful for public health interventions. Third, the population characteristics of our study are young and highly educated, which lacks generalizability.

## Conclusion

Our findings verified that the scale of solidarity among MSM could be useful in HIV testing promotion models. This study demonstrates that the community connectedness factor of an individual can influence the willingness of HIV testing during the epidemic. In addition, we found that the higher community connectedness MSM, who did not test for HIV, instead of those who had the test before the epidemic, were more willing to get the test. This study may help increase the HIV testing rate and reduce the burden of HIV amongst the MSM population.

## Data Availability Statement

The original contributions presented in the study are included in the article/Supplementary Material, further inquiries can be directed to the corresponding authors.

## Ethics Statement

Ethical approval was obtained from the Zhuhai Center for Diseases Control and Prevention. Potential participants clicked on the survey link and signed an electronic written consent inform and accepted quantification screening.

## Author Contributions

HL and WT: conceptualization, writing—original draft preparation, writing—review and editing, and visualization. HL and YZ: methodology. HL and SZ: software and validation. YZ, LZ, and LH: formal analysis. YZ, SH, LZ, WD, and HL: data curation. WD, LH, and WT: supervision. LH and WT: project administration and funding acquisition. All the authors have read and agreed to the published version of the manuscript.

## Conflict of Interest

The authors declare that the research was conducted in the absence of any commercial or financial relationships that could be construed as a potential conflict of interest.

## Publisher's Note

All claims expressed in this article are solely those of the authors and do not necessarily represent those of their affiliated organizations, or those of the publisher, the editors and the reviewers. Any product that may be evaluated in this article, or claim that may be made by its manufacturer, is not guaranteed or endorsed by the publisher.

## References

[1] Beyrer C Baral SD van Griensven F Goodreau SM Chariyalertsak S Wirtz AL. Global epidemiology of HIV infection in men who have sex with men. Lancet. (2012) 380:367–77. 10.1016/S0140-6736(12)60821-6 22819660 PMC3805037

[2] Sáez-Cirión A Sereti I. Immunometabolism and HIV-1 pathogenesis: food for thought. Nat Rev Immunol. (2021) 21 5–19. 10.1038/s41577-020-0381-7 32764670

[3] Zou H Hu N Xin Q Beck J. HIV testing among men who have sex with men in China: a systematic review and meta-analysis. AIDS Behav. (2012) 16:1717–28. 10.1007/s10461-012-0225-y 22677975

[4] Lui CW Dean J Mutch A Mao L Debattista J Lemoire J. HIV testing in men who have sex with men: a follow-up review of the qualitative literature since 2010. AIDS Behav. (2018) 22:593–605. 10.1007/s10461-017-1752-3 28331992

[5] Zhang TP Liu C Han L Tang W Mao J Wong T. Community engagement in sexual health and uptake of HIV testing and syphilis testing among MSM in China: a cross-sectional online survey. J Int AIDS Soc. (2017) 20:21372. 10.7448/IAS.20.01/21372 28406270 PMC5515028

[6] Tucker JD Muessig KE Cui R Bien CH Lo EJ Lee R. Organizational characteristics of HIV/syphilis testing services for men who have sex with men in South China: a social entrepreneurship analysis and implications for creating sustainable service models. BMC Infect Dis. (2014) 14:601. 10.1186/s12879-014-0601-5 25422065 PMC4247875

[7] Blanco JL Ambrosioni J Garcia F Martínez E Soriano A Mallolas J. COVID-19 in patients with HIV: clinical case series. Lancet HIV. (2020)7:e314–6. 10.1016/S2352-3018(20)30111-9 32304642 PMC7159872

[8] Jiang H Zhou Y Tang W. Maintaining HIV care during the COVID-19 pandemic. Lancet HIV. (2020) 7:e308–9. 10.1016/S2352-3018(20)30105-3 32272084 PMC7239666

[9] Hens K Nys H Cassiman JJ Dierickx K. Risks, benefits, solidarity: a framework for the participation of children in genetic biobank research. J Pediatr. (2011) 158:842–8. 10.1016/j.jpeds.2010.12.036 21349539

[10] McMillan DW. Sense of community. J Community Psycho. (1996) 24:315–25. Available online at: 10.1002/(sici)1520-6629(199610)24:4<315::aid-jcop2>3.0.co;2-t

[11] Frost DM Meyer IH. Measuring community connectedness among diverse sexual minority populations. J Sex Res. (2012) 49:36–49. 10.1080/00224499.2011.565427 21512945 PMC3143245

[12] Stirling K Toumbourou JW Rowland B. Community factors influencing child and adolescent depression: a systematic review and meta-analysis. Aust N Z J Psychiatry. (2015) 49:869–86. 10.1177/0004867415603129 26416916

[13] Ashmore RD Deaux K McLaughlin-Volpe T. An organizing framework for collective identity: articulation and significance of multidimensionality. Psychol Bull. (2004) 130:80–114. 10.1037/0033-2909.130.1.80 14717651

[14] Shigemoto Y Kawachi I. Social cohesion and quality of life among survivors of a natural disaster. Qual Life Res. (2020) 29:3191–200. 10.1007/s11136-020-02590-7 32696291

[15] Grover E Grosso A Ketende S Kennedy C Fonner V Adams D. Social cohesion, social participation and HIV testing among men who have sex with men in Swaziland. AIDS Care. (2016) 28:795–804. 10.1080/09540121.2015.1131971 26824888

[16] Painter TM Song EY Mullins MM Mann-Jackson L Alonzo J Reboussin BA. Social support and other factors associated with HIV testing by hispanic/latino gay, bisexual, and other men who have sex with men in the US South. AIDS Behav. (2019) 23:251–65. 10.1007/s10461-019-02540-6 31102108 PMC6800592

[17] Jiang H Xie Y Xiong Y Zhou Y Lin K Yan Y. HIV self-testing partially filled the HIV testing gap among men who have sex with men in China during the COVID-19 pandemic: results from an online survey. J Int AIDS Soc. (2021) 24:e25737. 10.1002/jia2.25737 34036750 PMC8150052

[18] Wu D Tang W Lu H Zhang TP Cao B Ong JJ. Leading by example: web-based sexual health influencers among men who have sex with men have higher HIV and syphilis testing rates in China. J Med Internet Res. (2019) 21:e10171. 10.2196/10171 30664490 PMC6360381

[19] Weikum D Shrestha R Ferro EG Vagenas P Copenhaver M Spudich S. An explanatory factor analysis of a brief self-report scale to detect neurocognitive impairment among HIV-positive men who have sex with men and transgender women in Peru. AIDS Care. (2017) 29:1297–301. 10.1080/09540121.2017.1322681 28449599 PMC6337065

[20] Paine EA Lee YG Vinogradov V Zhakupova G Hunt T Primbetova S. HIV stigma, homophobia, sexual and gender minority community connectedness and HIV testing among gay, bisexual, and other men and transgender people who have sex with men in kazakhstan. AIDS Behav. (2021) 25:2568–77. 10.1007/s10461-021-03217-9 33743115 PMC8424652

[21] Currin JM Giano Z Hubach RD. Interface of internalized homophobia and community connectedness on frequency of doctor's visits for rural and urban MSM in Oklahoma. J Rural Health. (2020) 36:416–22. 10.1111/jrh.12416 32057137

[22] Raymond HF Chen YH Stall RD McFarland W. Adolescent experiences of discrimination, harassment, connectedness to community and comfort with sexual orientation reported by adult men who have sex with men as a predictor of adult HIV status. AIDS Behav. (2011) 15:550–6. 10.1007/s10461-009-9634-y 19915973

[23] Holland CE Papworth E Billong SC Kassegne S Petitbon F Mondoleba V. Access to HIV services at non-governmental and community-based organizations among Men Who Have Sex with Men (MSM) in Cameroon: an integrated biological and behavioral surveillance analysis. PLoS ONE. (2015) 10:e0122881. 10.1371/journal.pone.0122881 25906046 PMC4408025

[24] Zhang W Hu Q Tang W Jin X Mao X Lu T. HIV self-testing programs to men who have sex with men delivered by social media key opinion leaders and community-based organizations are both effective and complementary: a national pragmatic study in China. J Acquir Immune Defic Syndr. (2020) 84:453–62. 10.1097/QAI.0000000000002375 32692103

[25] Tao J Li MY Qian HZ Wang LJ Zhang Z Ding HF. Home-based HIV testing for men who have sex with men in China: a novel community-based partnership to complement government programs. PLoS ONE. (2014) 9:e102812. 10.1371/journal.pone.0102812 25051160 PMC4106852

[26] Aronson ID Bennett AS Freeman R. Toward a human-centered use of technology: a stakeholder analysis of harm reduction and CBO staff. Harm Reduct J. (2020) 17:77. 10.1186/s12954-020-00422-y 33076911 PMC7570409

[27] Smith MK Stein G Cheng W Miller WC Tucker JD. Identifying high risk subgroups of MSM: a latent class analysis using two samples. BMC Infect Dis. (2019) 19:213. 10.1186/s12879-019-3700-5 30832592 PMC6399860

[28] Chuang DM Lacombe-Duncan A. Community engagement among men who have sex with men living with HIV/AIDS in Taiwan. AIDS Care. (2016) 28:445–9. 10.1080/09540121.2015.1112355 26586156

[29] Moran A Scheim A Lyons C Liestman B Drame F Ketende S. Characterizing social cohesion and gender identity as risk determinants of HIV among cisgender men who have sex with men and transgender women in Côte d'Ivoire. Ann Epidemiol. (2020) 42:25–32. 10.1016/j.annepidem.2019.11.003 31902624 PMC7205308

[30] Zhang TP Yang F Tang W Alexander M Forastiere L Kumar N. Pay-it-forward gonorrhea and chlamydia testing among men who have sex with men in China: a study protocol for a three-arm cluster randomized controlled trial. Infect Dis Poverty. (2019) 8:76. 10.1186/s40249-019-0581-1 31426869 PMC6700988

