# Supplementary material for: Solidarity and HIV Testing Willingness During the COVID-19 Epidemic: A Study Among Men Who Have Sex With Men in China
Source: Front Public Health. 2021 Dec 9;9:752965. doi: 10.3389/fpubh.2021.752965 (PMC8695798; doi:10.3389/fpubh.2021.752965)
Supplement: Supplementary file 1 [file Data_Sheet_1.docx]

Supplement table Hang Lyu, et al.

**Solidarity and HIV testing willingness during COVID-19 epidemic: A Study among Men who Have Sex with Men in China**

| Stable1. MSM cohesion（n=731） | | | | | | | | | | |
| --- | --- | --- | --- | --- | --- | --- | --- | --- | --- | --- |
| Variation | Total | % | Ever tested | Not tested | Variation | Number | % | Ever tested | Not tested |  |
| Do you think you are the one of the MSM group? (One_of_the_group) | | | | | If you need to borrow money, you think your friends in the MSM circle will lend it to you?(Borrow money) | | | | | |
| Very agree | 420 | 57.46 | 38.44 | 19.02 | Very agree | 112 | 15.32 | 10.67 | 4.65 |  |
| Agree | 287 | 39.26 | 24.90 | 14.36 | Agree | 306 | 41.86 | 27.77 | 14.09 |  |
| Don’t agree | 19 | 2.60 | 1.09 | 1.50 | Don’t agree | 235 | 32.15 | 19.70 | 12.45 |  |
| Strongly disagree | 5 | 0.68 | 0.41 | 0.27 | Strongly disagree | 78 | 10.67 | 6.70 | 3.97 |  |
| Your attitude towards join in the MSM community is positive?(Positive attitude join) | | | | | You are willing to discuss your private problems with friends in your MSM circle?(Privacy problem) | | | | | |
| Very agree | 348 | 47.61 | 32.56 | 15.05 | Very agree | 200 | 27.36 | 17.78 | 9.58 |  |
| Agree | 324 | 44.32 | 27.50 | 16.83 | Agree | 396 | 54.17 | 35.29 | 18.88 |  |
| Don’t agree | 52 | 7.11 | 4.24 | 2.87 | Don’t agree | 108 | 14.77 | 9.44 | 5.34 |  |
| Strongly disagree | 7 | 0.96 | 0.55 | 0.41 | Strongly disagree | 27 | 3.69 | 2.33 | 1.37 |  |
| You are pride of being part of the MSM group?(Pride group) | | | | | If you need a place to live, you can rely on your friends in your MSM circle to take you in?(Stay home) | | | | |  |
| Very agree | 201 | 27.50 | 17.37 | 10.12 | Very agree | 151 | 20.66 | 14.50 | 6.16 |  |
| Agree | 283 | 38.71 | 26.27 | 12.45 | Agree | 369 | 50.48 | 32.01 | 18.47 |  |
| Don’t agree | 217 | 29.69 | 19.43 | 10.26 | Don’t agree | 176 | 24.08 | 15.05 | 9.03 |  |
| Strongly disagree | 30 | 4.10 | 1.78 | 2.33 | Strongly disagree | 35 | 4.79 | 3.28 | 1.50 |  |
| You think that as long as you work with your partner, the problems facing the MSM community can be solved?(Solve effort) | | | | | Do you think your MSM circle is a harmonious circle?(Harmony circle) | | | | | |
| Very agree | 209 | 28.59 | 19.84 | 8.76 | Very agree | 110 | 15.05 | 9.71 | 5.34 |  |
| Agree | 309 | 42.27 | 27.50 | 14.77 | Agree | 353 | 48.29 | 33.11 | 15.18 |  |
| Don’t agree | 180 | 24.62 | 14.91 | 9.71 | Don’t agree | 225 | 30.78 | 18.74 | 12.04 |  |
| Strongly disagree | 33 | 4.51 | 2.60 | 1.92 | Strongly disagree | 43 | 5.88 | 3.28 | 2.60 |  |
| Do you think the problems faced by any MSM community are also problems faced by you?(Same problems) | | | | | You can trust most MSM you know?(Trust MSM) | | | | | |
| Very agree | 277 | 37.89 | 26.27 | 11.63 | Very agree | 110 | 15.05 | 9.44 | 5.61 |  |
| Agree | 366 | 50.07 | 31.87 | 18.19 | Agree | 349 | 47.74 | 33.11 | 14.64 |  |
| Don’t agree | 79 | 10.81 | 5.75 | 5.06 | Don’t agree | 233 | 31.87 | 19.56 | 12.31 |  |
| Strongly disagree | 9 | 1.23 | 0.96 | 0.27 | Strongly disagree | 39 | 5.34 | 2.74 | 2.60 |  |

Ever tested: MSM who had ever tested for HIV before the COVID-19 epidemic(2020-1-25); Not tested: MSM who didn’t test for HIV before the epidemic(2020-1-25).

Stable2. Distribution of HIV testing and self-test reagent usage before and during the epidemic（n=731）

| Variation | Number(%) |
| --- | --- |
| **Before the epidemic** |  |
| Have you ever been tested for HIV |  |
| Yes | 474 (64.84) |
| No | 257 (35.16) |
| Whether to use self-check reagents（n=474） |  |
| Yes | 389 (82.07) |
| No | 85 (17.93) |
| **During the epidemic** |  |
| Do you want to be tested for HIV |  |
| Yes | 427 (58.41) |
| No | 304 (41.59) |
| Does HIV testing meet the needs（n=427） |  |
| Yes | 277 (64.87) |
| No | 150 (35.13) |
| Whether to use self-check reagents（n=277） |  |
| Yes | 233 (84.12) |
| No | 44 (15.88) |


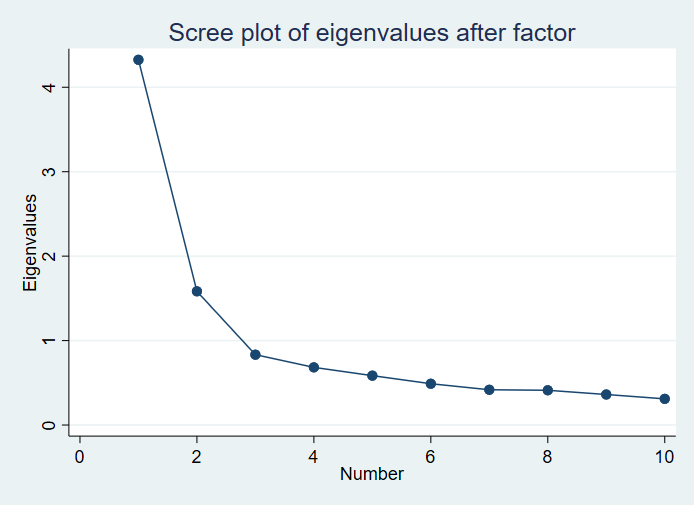


SFigure1. Factor analysis
